# A short hepatitis C virus NS5A peptide expression by AAV vector modulates human T cell activation and reduces vector immunogenicity

**DOI:** 10.1038/s41434-021-00302-5

**Published:** 2021-11-11

**Authors:** Winston Colon-Moran, Alan Baer, Gauri Lamture, Jack T. Stapleton, Joseph W. Fischer, Nirjal Bhattarai

**Affiliations:** 1 Division of Cellular and Gene Therapies, Office of Tissues and Advanced Therapies, Center for Biologics Evaluation and Research, Food and Drug Administration, Silver Spring, MD 20993 USA; 2 Research Service, Iowa City Veterans Affairs Medical Center, Iowa City, IA USA; 3 Departments of Internal Medicine and Microbiology, University of Iowa, Iowa City, IA USA; 4 Present Address: Adicet Bio, Inc., Menlo Park, CA USA; 5 Present Address: AstraZeneca, Gaithersburg, MD USA

**Keywords:** Adaptive immunity, Virology

## Abstract

Viral vector-mediated gene therapies have the potential to treat many human diseases; however, host immune responses against the vector and/or the transgene pose a safety risk to the patients and can negatively impact product efficacy. Thus, novel strategies to reduce vector immunogenicity are critical for the advancement of these therapies. T cell activation (TCA) is required for the development of immune responses during gene therapy. We hypothesized that modulation of TCA by incorporating a novel viral immunomodulatory factor into a viral vector may reduce unwanted TCA and immune responses during gene therapy. To test this hypothesis, we identified an immunomodulatory domain of the hepatitis C virus (HCV) NS protein 5A (NS5A) protein and studied the effect of viral vectors expressing NS5A peptide on TCA. Lentiviral vector-mediated expression of a short 20-mer peptide derived from the NS5A protein in human T cells was sufficient to inhibit TCA. Synthetic 20-mer NS5A peptide also inhibited TCA in primary human T cells. Mechanistically, the NS5A protein interacted with Lck and inhibited proximal TCR signaling. Importantly, NS5A peptide expression did not cause global T cell signaling dysfunction as distal T cell signaling was not inhibited. Finally, recombinant adeno-associated virus (AAV) vector expressing the 20-mer NS5A peptide reduced both the recall antigen and the TCR-mediated activation of human T cells and did not cause global T cell signaling dysfunction. Together, these data suggest that expression of a 20-mer NS5A peptide by an AAV vector may reduce unwanted TCA and may contribute to lower vector immunogenicity during gene therapy.

## Introduction

Recombinant adeno-associated virus (AAV) vectors are commonly used as gene therapy delivery systems. Currently, there are two US Food and Drug Administration (FDA)-approved gene therapies that use AAV vector in vivo [1]. Although viral vector-mediated in vivo gene therapies (VVIGTs) are showing potential to treat various human diseases [2–4], several challenges limit their widespread use [5]. Pre-existing immunity and de novo immune responses against the viral vector and transgene product can limit the successful clinical translation of VVIGTs [6]. Many preclinical and clinical studies have demonstrated activation of both the innate and the adaptive immune responses during gene therapy with common viral vectors derived from AAV, adenovirus, and lentivirus [7–10]. The host immune response against these viral vectors poses not only a safety risk to subjects receiving these therapies but can also reduce treatment efficacy and limit re-treatment options. While numerous strategies have been proposed to reduce host immune response during VVIGTs [11]; to date, the safety and efficacy of most of these strategies in humans remain unknown.

Currently, immunosuppressive (IS) drugs are commonly used to dampen host immune responses during gene therapy [12, 13]. These IS drugs have a systemic effect on the immune system and typically cause unwanted effects [14–16]. Furthermore, IS drugs have a heterogeneous response in the human population [17], and there are concerns over potential risks associated with the use of IS drugs in certain patient subpopulations such as immunocompromised people who may benefit from gene therapy. In addition, vector- and transgene-specific immune responses may arise after cessation of IS drug treatment [13, 18, 19]. These challenges impede the development of gene therapies for many diseases affecting diverse human populations. Thus, developing novel strategies to reduce host immune response against viral vectors during gene therapy is essential to overcome these limitations and help advance these therapies.

T cell receptor (TCR)-mediated T cell activation (TCA) is an essential step in generating both the cellular and the humoral immune response [20]. Many viruses have evolved to interfere with TCR signaling and inhibit TCA to evade immune responses and establish persistent infection [21]. Among human RNA viruses, the hepatitis C virus (HCV) has a remarkable ability to cause persistent infection. HCV has evolved to inhibit both the innate and the adaptive immune responses by various mechanisms [22, 23]. The HCV core protein and the envelope protein (E2) inhibit TCA and the nonstructural (NS) proteins 3/4A and 5A inhibit the innate immune response [24–30].

The HCV NS protein 5A (NS5A) protein is a multifunctional phosphoprotein and plays an important role in viral replication [25]. Although previous studies have identified a role of NS5A in modulating innate immune response [25, 31, 32], its effect on human TCA has not been studied. Here, we studied the effect of HCV NS5A protein on human TCR signaling pathways and TCA. We identified a short 20-amino-acid (a.a.) domain within the HCV NS5A protein that inhibits TCA. Furthermore, we identified the mechanism by which HCV NS5A inhibits TCA. Finally, we assessed the effect of a recombinant AAV vector expressing the 20-mer NS5A peptide on human TCR signaling pathways and TCA.

## Results

### HCV NS5A expression inhibits TCR-mediated activation of human T cells

**Fig. 1:**
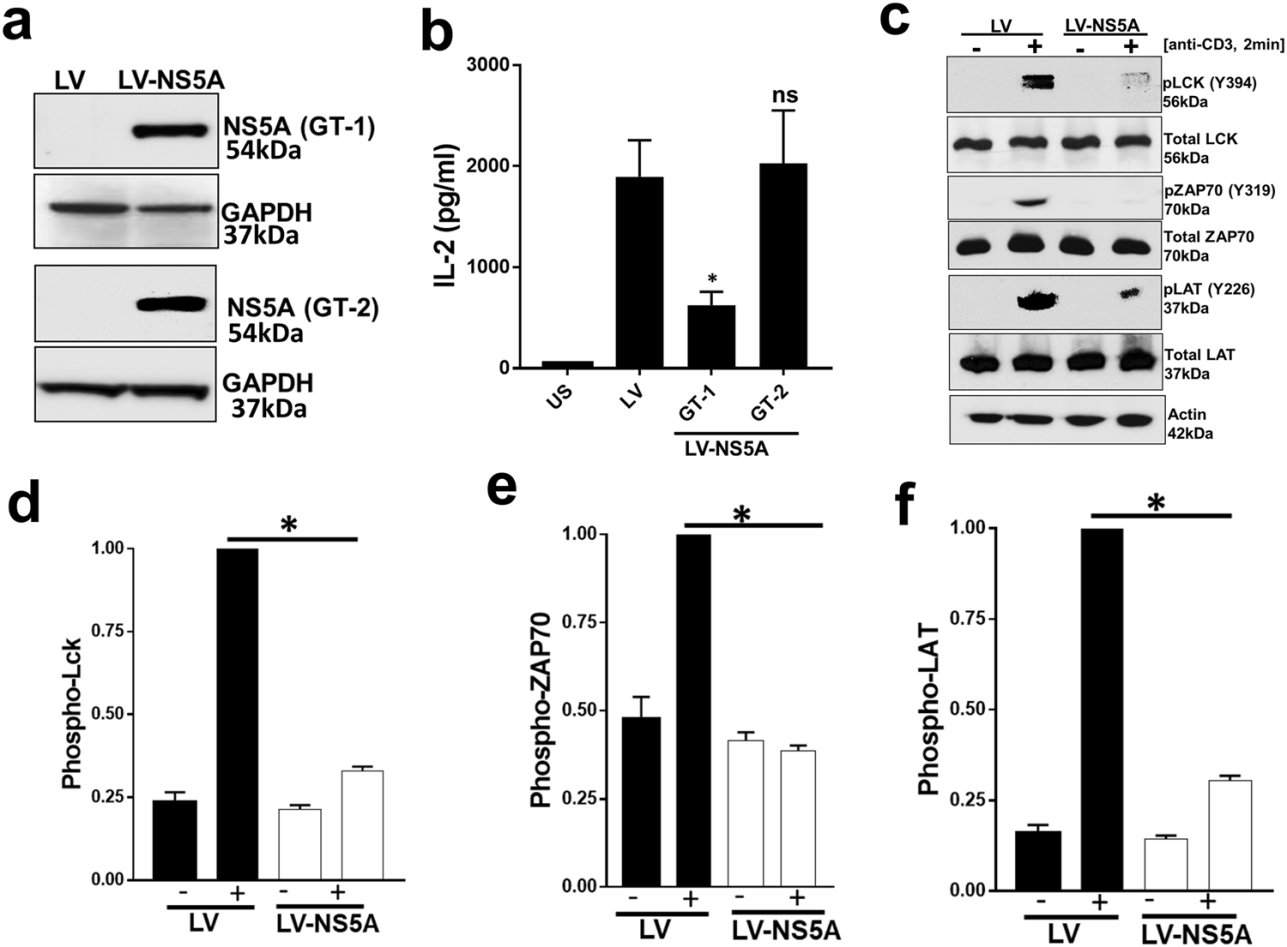
HCV NS5A expression inhibits TCR-mediated activation of human T cells. **a** Immunoblot analysis of Jurkat T cells transduced with a control lentiviral vector (LV) or a lentiviral vector expressing hepatitis C virus (HCV) NS5A protein (LV-NS5A) from genotype 1 (GT-1) or genotype 2 (GT-2). GAPDH expression was measured as a loading control. **b** Following TCR engagement with anti-CD3/CD28, T cell activation was measured by assessing IL-2 release. **c** Activation of proximal TCR signaling events was measured in LV- or LV-NS5A-transduced Jurkat cells. Cells were either unstimulated (−) or anti-CD3 stimulated (2 min, +) and phosphorylation of Lck at tyrosine 394 (pY394), ZAP-70 at pY319, and LAT at pY226 were assessed. Phosphorylation of both 56 and 61 kDa Lck isoforms were detected. Actin protein expression was measured as a loading control. Quantification of immunoblots for **d** phospho-Lck, **e** phospho-ZAP-70, and **f** phospho-LAT obtained from the independent experiments are shown. Phosphoprotein levels were normalized to actin. Data represent the average of three independent experiments, and the standard deviation is shown. *P < 0.01.

To assess the effect of HCV NS5A on TCA, a human CD4^+^ T cell line (Jurkat) was transduced with a control lentiviral vector (LV) or LV encoding the NS5A protein from two different HCV genotypes (GTs), GT-1 or GT-2. Expression of the NS5A protein from both HCV GTs was detected in Jurkat cells by immunoblot analysis (Fig. 1a). Following TCR engagement with anti-CD3/CD28, TCA was measured by assessing interleukin-2 (IL-2) release. Interestingly, the expression of NS5A protein from GT-1 but not GT-2 resulted in inhibition of TCR-mediated TCA compared to Jurkat cells transduced with control LV expressing green fluorescent protein (GFP) alone (Fig. 1b). There are seven GTs of HCV [33] and NS5A protein has considerable differences in amino acid sequence among HCV GTs [34]. The observation that the NS5A protein from GT-1 but not GT-2 inhibited TCA prompted us to further assess both proteins to better understand the mechanism by which NS5A (GT-1) inhibits TCA.

Engagement of the TCR with peptide-bound major histocompatibility complex present on the surface of antigen-presenting cells results in activation of intracellular T cell signaling events resulting in TCA. One of the earliest events following TCR engagement is the activation of lymphocyte-specific tyrosine kinase (Lck) [35]. In resting T cells, Lck is phosphorylated at the carboxy-terminal tyrosine residue (Y505) by the C-terminal Src kinase [36–38]. Lck phosphorylated at the carboxy-terminal tyrosine maintains a closed conformation and is enzymatically inactive [38–40]. Upon TCR engagement Lck is dephosphorylated resulting in a conformational change that allows trans-autophosphorylation of the tyrosine residue (Y394) in the kinase domain [36, 38–40]. Lck phosphorylated at the Y394 maintains an open conformation, is enzymatically active, and mediates downstream TCR signaling [36, 38, 41–44].

Since the expression of the NS5A protein from HCV (GT-1) inhibited TCR-mediated TCA, we next assessed the effect of NS5A expression on Lck activation. Following anti-CD3 mediated TCR engagement, activation of Lck was assessed by immunoblots in Jurkat cells transduced with either control LV or LV-NS5A encoding HCV (GT-1) NS5A protein. Activation of Lck, as measured by phosphorylation at Y394, was significantly reduced in Jurkat cells transduced with the LV-NS5A compared to the control LV (Fig. 1c, d). Phosphorylation observed in T cells transduced with control LV was considered as a positive control. Active Lck phosphorylates and activates ZAP-70 tyrosine kinase, which then phosphorylates adaptor protein LAT, theh linker of activated T cells [36, 45]. Phosphorylated LAT protein plays a key role in proximal TCR signaling by forming a multiprotein complex that activates downstream signaling factors [45]. We found that HCV (GT-1) NS5A expression reduced phosphorylation of both ZAP-70 (Fig. 1c, e) and LAT (Fig. 1c, f) following TCR engagement. Together, these data suggest that HCV (GT-1) NS5A protein inhibits proximal (upstream) TCR signaling by reducing Lck activation.

### HCV (GT-1) NS5A interacts with Lck in human T cells

**Fig. 2:**
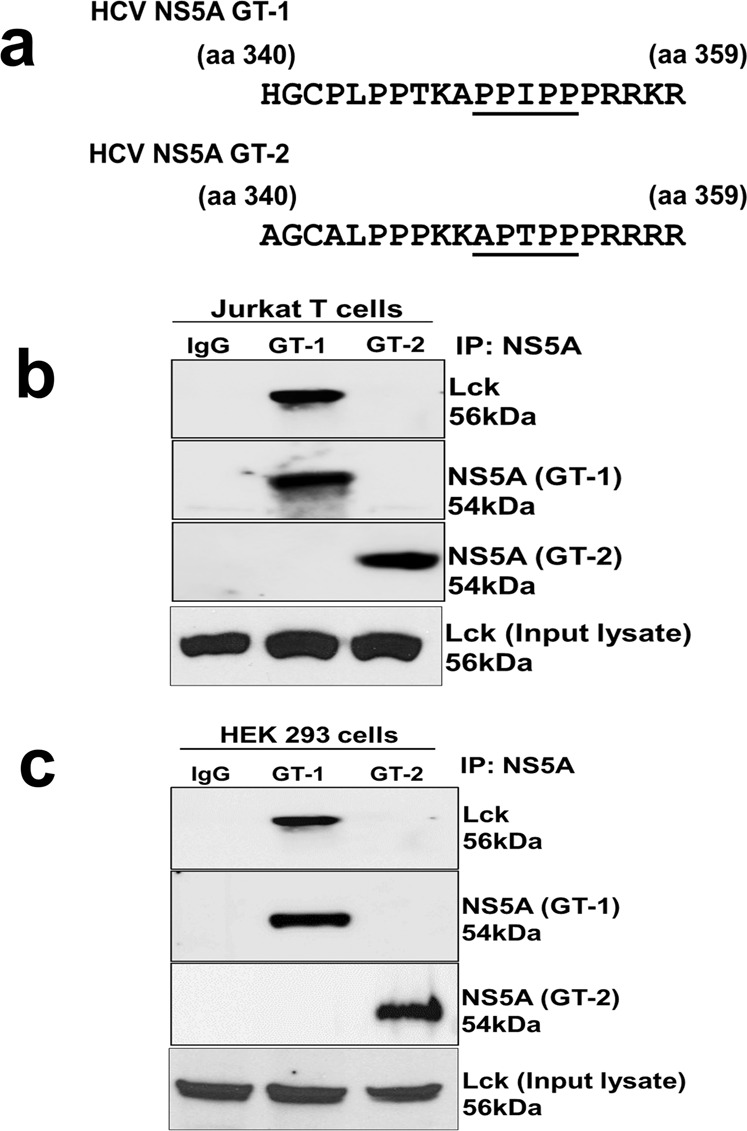
HCV (GT-1) NS5A interacts with Lck in human T cells. Panel **a** illustrates a peptide region of HCV NS5A protein (amino acids 340–359) from two genotypes (GT-1 and GT-2) that contains the poly-proline-rich motif (underlined). HCV NS5A protein was immunoprecipitated from **b** Jurkat T cells or **c** HEK 293 cells expressing either NS5A, GT-1 or GT-2, and Lck. The precipitates were analyzed by immunoblots using anti-Lck and anti-NS5A antibodies. Two different antibodies were used to detect NS5A proteins from genotype 1 and genotype 2 as described in the “Methods” section. Isotype matching antibodies were used as a negative control during immunoprecipitation. A representative immunoblot analysis is shown. Each experiment was independently performed at least three times with similar results.

HCV NS5A is known to interact with numerous cellular factors [25]. A previous study found that HCV (GT-1) NS5A interacts with various members of the Src-family kinases, including Lck in nonhuman epithelial cell line (COS-7) via the poly-proline-rich motif (PPRM) located at the C terminus of the NS5A protein (a.a. 340–359) [46] (Fig. 2a). The PPRM motif contains a minimal consensus sequence, proline-X-X-proline (PXXP, a.a. 350–353) (Fig. 2a, underlined), that interacts with proteins containing the Src-homology 3 domain (SH3 domain) such as Lck [47, 48]. In a previous study, the PPRM motif of HCV (GT-1) NS5A was found to be essential for interaction with the SH3 domain of Lck, and mutation of the proline residues at positions 350, 353, and 354 to alanine within the PXXP motif prevented NS5A and Lck interactions [46]. A sequence analysis revealed that the NS5A protein from HCV (GT-2) contained an alanine (A) at position 350 instead of a proline, resulting in the formation of AXXP instead of PXXP (Fig. 2a, underlined). Since we found that the NS5A from HCV GT-1, but not GT-2 inhibited TCA (Fig. 1b) and the NS5A (GT-2) contained an alanine at position 350 instead of a proline, we hypothesized that NS5A from GT-1 but not GT-2 interacts with Lck in human cells due to this mutation. To test this hypothesis, NS5A protein from both HCV GTs were expressed in Jurkat T cells and HEK 293 cells. Following immunoprecipitation of NS5A, its interaction with Lck was assessed by immunoblotting. We found that NS5A from HCV (GT-1) interacted with Lck in both Jurkat T cells (Fig. 2b) and HEK 293 cells (Fig. 2c); however, in both cell lines, NS5A from HCV (GT-2) did not interact with Lck. Together, these data suggest that the NS5A protein from HCV GT-1 but not GT-2 interacts with Lck.

### Expression of a short HCV (GT-1) NS5A peptide inhibits TCR-mediated activation of human T cells

**Fig. 3:**
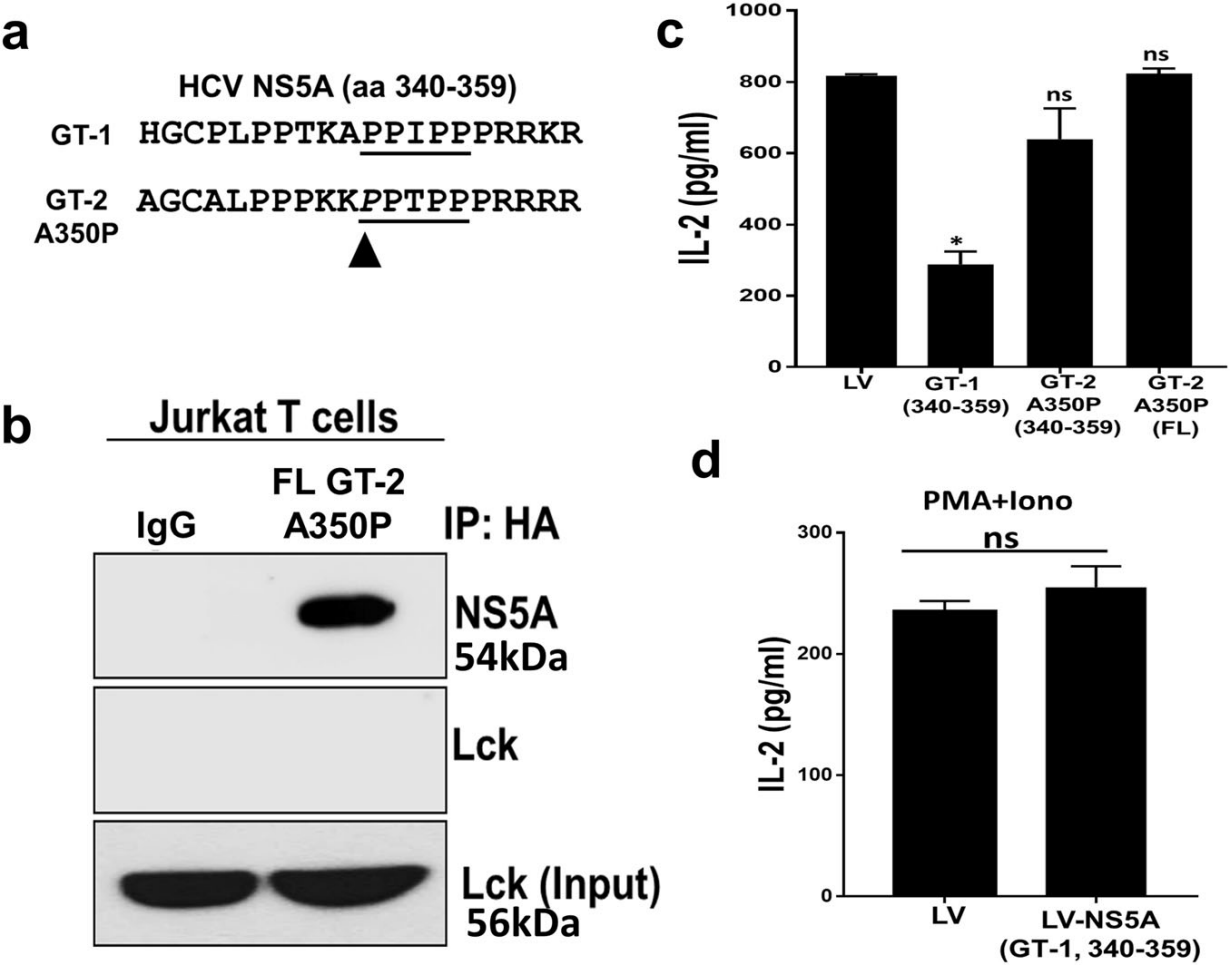
Expression of a short HCV (GT-1) NS5A peptide inhibits TCR-mediated activation of human T cells. Panel **a** illustrates a peptide region of NS5A protein (amino acids, a.a. 340–359) from GT-1 and GT-2 that contains the poly-proline-rich motif (underlined). HCV NS5A (GT-2) was mutated to contain a proline instead of alanine at position 350 (A350P, arrow). **b** HCV NS5A protein (GT-2) containing an alanine-to-proline mutation at position 350 (A350P) was immunoprecipitated from Jurkat T cells using anti-HA antibodies that react to a C-terminal HA-tag present in the NS5A protein. The precipitates were analyzed by immunoblots using anti-Lck and anti-HA antibodies. Isotype matching antibody was used as a negative control during immunoprecipitation. The input cell lysate used for immunoprecipitation was also assessed for Lck expression. A representative immunoblot analysis is shown and was independently performed at least three times with similar results. **c** Following TCR engagement with anti-CD3/CD28, T cell activation was measured by assessing IL-2 release in Jurkat cells transduced with a control lentiviral vector (LV) or LV expressing a short peptide (a.a. 350–359) derived from NS5A protein GT-1 or GT-2 with A350P mutation or LV expressing a full-length (FL) NS5A protein of HCV (GT-2) containing A350P mutation. **d** Following PMA/ionomycin stimulation, T cell activation was measured in Jurkat T cells transduced with a control lentivirus vector (LV) or LV expressing NS5A (GT-1) peptide (a.a. 340–359). IL-2 was normalized to levels released by cells transduced with control LV. Data represent the average of three technical replicates and the standard deviation is shown. Each experiment was repeated at least three times with similar results. *P < 0.01; n.s. not significant.

As noted above, HCV (GT-1) NS5A contains the PPRM at the C terminus, which forms a minimal consensus sequence, PXXP required for its interaction with Lck [46]. To assess if the expression of the PPRM motif is sufficient to inhibit TCR-mediated TCA, an LV encoding a short 20 a.a. peptide domain of the HCV (GT-1) NS5A protein (a.a. 340–359) that contains the PPRM motif was generated (Fig. 3a). Furthermore, LVs encoding either the full-length (FL) HCV (GT-2) NS5A or the 20-mer peptide containing an alanine to proline mutation at position 350 (A350P), to restore the PXXP motif, were also generated to assess if restoring PXXP motif is sufficient for interactions between NS5A (GT-2) and Lck (Fig. 3a, underlined).

Jurkat cells were transduced with all LVs and transduction was assessed by analyzing GFP produced by the internal ribosomal entry site present within the vector. All cell lines expressed GFP as measured by flow cytometry (Supplemental Fig. 1). Interestingly, restoration of the PXXP motif with A350P mutation in the NS5A (GT-2) protein did not rescue its interaction with Lck in human T cells (Fig. 3b). This suggests that mutation of alanine at position 350 to proline is not sufficient to restore interactions between HCV (GT-2) NS5A and Lck and that other residues outside the PXXP motif are necessary for this interaction.

Next, we assessed the effect of 20-mer NS5A peptide expression on TCR-mediated TCA. Following TCR engagement with anti-CD3/CD28, TCA was measured by assessing IL-2 release. Expression of the 20-mer HCV (GT-1) NS5A peptide significantly inhibited TCA, suggesting that the 20-mer peptide derived from NS5A (GT-1) was sufficient to inhibit TCA (Fig. 3c). Similar to FL NS5A protein expression, 20-mer NS5A peptide expression also inhibited proximal TCR signaling pathways as measured by phosphorylation of Lck, ZAP-70 and LAT (Supplemental Fig. 2). These results suggest that both the FL NS5A protein and the 20-mer NS5A peptide inhibit TCA by inhibiting activation of proximal TCR signaling events.

Neither the FL NS5A protein nor the 20-mer peptide from HCV (GT-2) containing the A350P mutation inhibited TCR-mediated TCA compared to control LV-transduced cells (Fig. 3c). This further demonstrates that restoration of the PXXP motif in the NS5A protein from HCV (GT-2) is not sufficient for inhibition of TCA.

To ensure that expression of the 20-mer NS5A peptide from HCV (GT-1) did not cause global T cell signaling dysfunction, TCA was assessed following treatment with phorbol 12-myristate 13-acetate (PMA) and ionomycin. Activation with PMA and ionomycin bypasses activation of the proximal TCR signaling events and activates distal (downstream) T cell signaling pathways through activation of protein kinase C and calcineurin [49]. The 20-mer NS5A peptide from HCV GT-1 did not inhibit PMA/ionomycin-induced TCA (Fig. 3d), suggesting that NS5A peptide expression does not cause global T cell signaling dysfunction.

### Synthetic HCV (GT-1) NS5A peptide inhibits TCR-mediated activation of primary human T cells

**Fig. 4:**
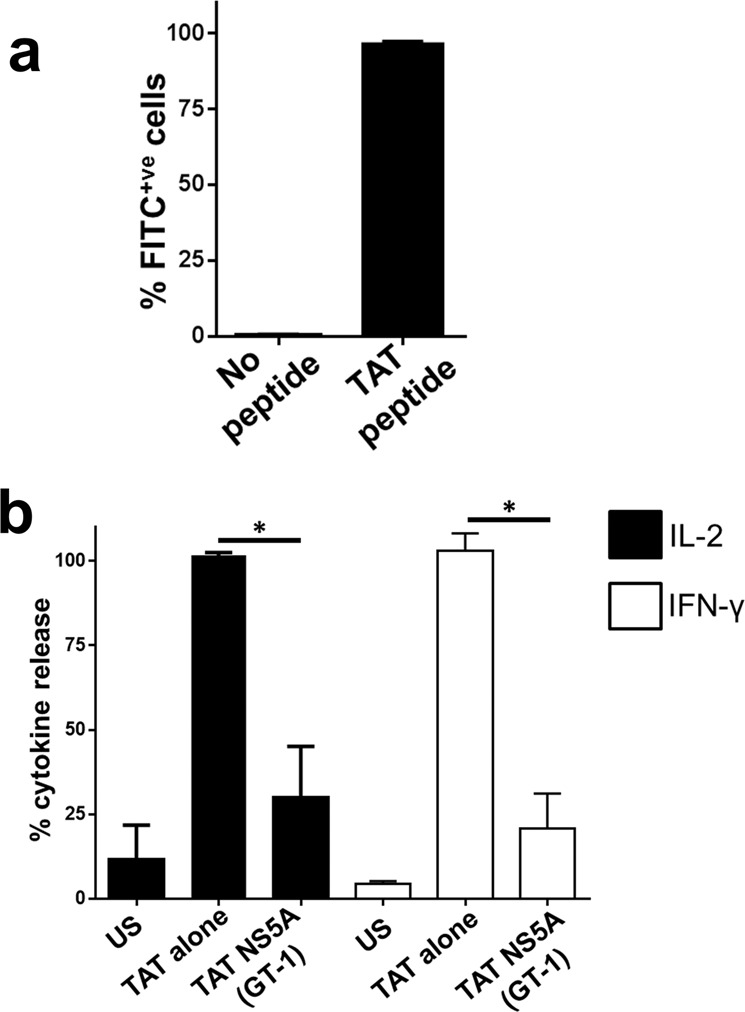
Synthetic HCV (GT-1) NS5A peptide inhibits TCR-mediated activation of primary human T cells. **a** Flow cytometry analysis of primary human T cells following 24-h incubation with FITC-labelled synthetic peptide. Cells were either left untreated (No peptide) or treated with a peptide with an HIV TAT protein transduction domain sequence at the N terminus (TAT peptide). Each experiment was conducted in triplicate and repeated on a separate day with consistent results. **b** Primary human T cells were treated with TAT control peptides or peptides representing HCV NS5A (GT-1) sequence (a.a. 340–359). Following TCR engagement with anti-CD3, T cell activation was measured by assessing IL-2 and IFN-γ release. Data represent the average of three technical replicates, and the standard deviation is shown. Each experiment was independently performed using three different donors with similar results. *P < 0.01; US unstimulated.

Although the data obtained from Jurkat T cells strongly suggest that expression of the 20-mer NS5A peptide from HCV (GT-1) results in inhibition of TCR-mediated TCA, we sought to confirm this observation by assessing the effect of synthetic NS5A peptide on TCR-mediated activation of primary human T cells. Synthetic NS5A peptide (HGCPLPPTKAPPIPPPRRKR) representing a.a. 340–359 of the HCV (GT-1) NS5A was generated with an N-terminal HIV TAT protein transduction domain (TAT; GGGGGRKKRRQRRR) to enhance cellular uptake (TAT-GT-1). A control peptide containing the TAT domain alone was also synthesized (TAT alone). Peptides were also labeled with N-terminal fluorescein isothiocyanate (FITC) tag to monitor cellular uptake. Primary human T cells were treated with FITC-labelled synthetic peptides and following 18 h of incubation cellular uptake was assessed by flow cytometry. Compared to untreated cells, FITC-peptide treated cells were FITC-positive confirming cellular uptake (Fig. 4a). Following anti-CD30mediated TCR engagement, TCA was measured by assessing IL-2 and interferon-gamma (IFN-γ) release. Treatment of primary human T cells with synthetic NS5A (GT-1) peptide significantly reduced TCR-induced IL-2 and IFN-γ release compared to the control (TAT alone) peptide (Fig. 4b). These data confirm that the 20-mer peptide (a.a. 340–359) derived from the HCV (GT-1) NS5A protein is sufficient to inhibit TCR-mediated TCA of primary human T cells.

### Recombinant AAV vector expressing the 20-mer NS5A peptide inhibits TCA

**Fig. 5:**
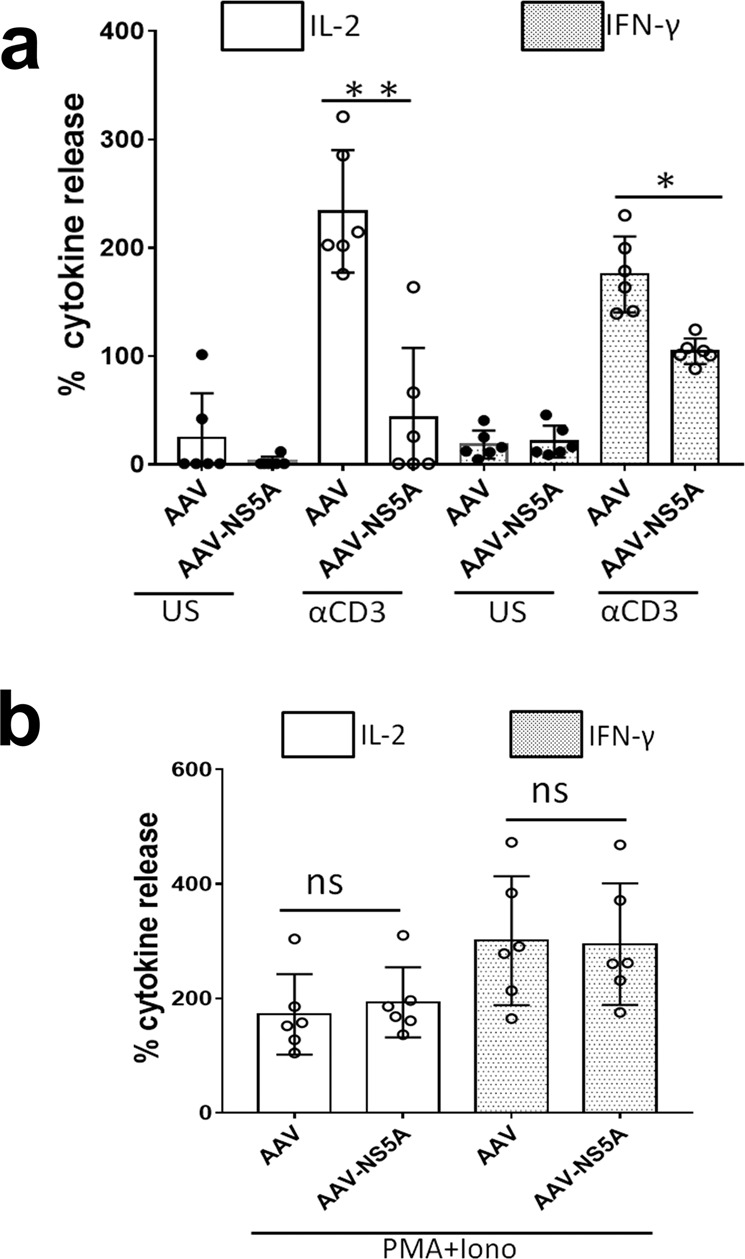
Recombinant AAV vector expressing the 20-mer NS5A peptide inhibits TCA. Primary human T cells were either mock transduced or transduced with a control AAV vector or a vector expressing HCV NS5A (GT-1) peptide (340–359) (AAV-NS5A). Transduced cells were either unstimulated (US) or stimulated with **a** anti-CD3 or **b** PMA/ionomycin and T cell activation was measured by assessing IL-2 and IFN-γ release. Cytokine levels were normalized to cytokines released by mock-transduced T cells following activation. Data represent the average from six donors and the standard deviation is shown. **P < 0.0001; *P < 0.001; n.s. not significant.

Recombinant AAV vector has limited packaging capacity. Thus, incorporation of large immunomodulatory factor(s) to reduce vector immunogenicity can limit the transgene packaging capacity of the vector. Since we identified a short 20-mer NS5A peptide that can inhibit the proximal TCR signaling and activation of primary human T cells, we sought to test the ability of a recombinant AAV vector (serotype 6) encoding this short peptide to modulate TCA ex vivo. Primary human T cells from healthy donors were incubated either with the control AAV vector encoding GFP (AAV) or encoding NS5A peptide and GFP (AAV-NS5A). After 48 h, T cells were activated using anti-CD3 and TCA was assessed by measuring IL-2 and IFN-γ released in the culture supernatant. In the absence of anti-CD3 stimulation (unstimulated), IL-2 was detected in T cells from two out of six donors incubated with the control AAV vector but was not detected in cells from the same donors incubated with AAV-NS5A. In the absence of CD3 stimulation, IL-2 release is likely due to activation of pre-existing memory T cells in these donors. This suggests that AAV vector expressing NS5A peptide may also inhibit recall antigen-mediated T cell response (Fig. 5a). Following anti-CD3-mediated TCR engagement, IL-2 and IFN-γ were detected in T cells obtained from all donors that were transduced with the control AAV vector (Fig. 5a). However, TCR-stimulated IL-2 and IFN-γ were significantly reduced in T cells from the same donors that were transduced with AAV-NS5A vector. This is consistent with previous findings (Figs. 3 and 4) and demonstrates that expression of a short NS5A peptide of HCV (GT-1) either via an LV or an AAV vector inhibits TCA in human T cells.

To ensure that AAV-NS5A does not cause global dysfunction of T cell signaling pathways, control AAV or AAV-NS5A-transduced T cells were also stimulated with PMA and ionomycin (PMA + Iono). Consistent with the previous observation (Fig. 3d), AAV-NS5A did not inhibit PMA + Iono-mediated TCA as measured by IL-2 and IFN-γ release, suggesting that the NS5A peptide expression does not cause global T cell signaling dysfunction (Fig. 5b), but rather acts in a targeted manner affecting proximal/upstream signaling events.

## Discussion

VVIGT is rapidly evolving, and many new therapies are currently being developed. Many of these therapies likely will reach clinical studies with the promise of curing previously incurable human diseases. One of the major challenges with viral vectors is the induction of host immune responses against the vector and the transgene product. Currently, IS drugs are commonly used to mitigate host immune responses during gene therapy, but IS drugs have numerous unwanted systemic effects and can have deleterious side effects both for the patient and the therapeutic vector [50]. These drugs also tend to have temporal effects and the immune response particularly to transgene may arise after drug cessation. Thus, novel strategies to favorably modulate the host immune responses are warranted.

In this study, we characterized the effect of HCV NS5A on human TCA signaling pathways with the goal to identify novel, viral-based strategies to modulate host T cell responses during gene therapy. As previous studies had identified immunomodulatory functions of the HCV NS5A protein [31, 46], we sought to further characterize these immunomodulatory effects on human TCA, with the potential to apply this to gene therapy vectors. We found that LV expressing the FL HCV NS5A protein from GT-1 but not from GT-2 inhibited proximal TCR-mediated activation of human T cells (Fig. 1). Lck as well as ZAP-70 and LAT are proximal T cell signaling molecules that are phosphorylated and activated following TCR engagement [51]; it is also known that Lck is targeted by numerous viruses to inhibit TCA [52]. We found that the NS5A (GT-1) protein expression resulted in inhibition of proximal TCR signaling pathways in part due to inhibition of Lck activation and its downstream targets ZAP-70 and LAT. The inhibition of proximal TCR signaling pathways resulted in inhibition of IL-2 release from Jurkat cells expressing NS5A (GT-1) (Fig. 1b). Although we did not measure IL-2 transcripts in these cells, IL-2 is transcribed only after TCA, which is translated and released from the cells. As we found that GT-1 NS5A protein inhibits TCA, IL-2 transcripts are likely also inhibited, as our previous work demonstrated that IL-2 transcripts are reduced along with IL-2 protein levels following inhibition of TCA pathways [53].

To better understand the mechanism for Lck inactivation by NS5A (GT-1), we also assessed interactions between NS5A and Lck protein. A previous study found that a PPRM of the NS5A from HCV GT-1 binds to the SH3 domain-containing proteins including Lck [46]. We found that NS5A from GT-1 but not GT-2 interacted with Lck, suggesting that the HCV GT-1 NS5A binds to Lck and inhibits its activation following TCR engagement. Sequence analysis revealed that the PPRM of the NS5A GT-1 has a consensus SH3 domain-binding motif (PXXP); however, instead of a proline at position 350, the NS5A protein of HCV GT-2 has an alanine resulting in AXXP, a non-consensus sequence. To test if the alanine residue at position 350 is responsible for the inability of NS5A (GT-2) to interact with Lck, alanine was mutated to proline (A350P) to restore the PXXP motif in the NS5A from GT-2. However, neither the interactions with Lck nor the reduction in TCA was observed with the mutant NS5A (GT-2) protein containing A350P. This suggests that other residues outside the PPRM or other differences between GT-1 and GT-2 may be responsible for interactions with Lck. Lack of interactions between NS5A GT-2 and Lck may explain why NS5A GT-2 does not inhibit TCA; however, further studies are necessary to better understand the relationship between NS5A protein from different HCV GTs and its effect on TCA.

Our results indicate that the expression of a short 20 a.a. (20-mer) peptide of HCV NS5A (GT-1) is sufficient to inhibit TCA in primary human T cells (Figs. 3 and 4). We also found that recombinant AAV vector expressing the 20-mer peptide inhibits primary human TCA ex vivo (Fig. 5), suggesting that incorporation of short 20-mer HCV peptide may help reduce unwanted TCA during gene therapy. In this study, a recombinant AAV vector containing capsid derived from AAV serotype 6, which efficiently transduces T cells, was used to assess the ability of a short NS5A peptide to modulate T cell function. For AAV vectors that do not transduce T cells or if the target cells of AAV vectors are non-T cells, NS5A peptide expression in the target cells alone may not be sufficient to modulate vector immunogenicity. In those cases, strategies such as engineering AAV vector to secrete NS5A peptide, so that secreted peptide will be taken up by bystander T cells, would be necessary to modulate T cell responses.

One of the limitations of AAV vector is its packaging capacity; thus, incorporation of any additional factor can limit transgene packaging capacity. Furthermore, the incorporation of larger proteins or peptides may also inherently induce an immune response. Thus, the use of a smaller peptide, such as the 20-mer peptide identified in our study has an advantage over the expression of larger and potentially antigenic proteins or peptides identified in other studies to modulate host immune response against AAV vector [54].

In conclusion, the immunogenicity of gene therapy products is a significant challenge that needs to be addressed by developing novel immunomodulatory approaches. Our study has identified a novel strategy to dampen the host T cell response during AAV-mediated gene therapy. Current studies are underway to assess the potential of the 20-mer NS5A peptide to modulate the immune response against AAV vector in vivo.

## Methods

### Peripheral blood mononuclear cells (PBMCs)

Whole blood was obtained from healthy donors at the National Institutes of Health (NIH) Blood Bank. All the donors provided written consent for their blood products to be used in research projects. All the samples provided to the investigators were de-identified. This study was exempted by the FDA’s IRB, Research Involving Human Subject Committee (RIHSC). PBMCs were isolated from whole blood using Ficoll-Hypaque gradient centrifugation. Isolated PBMCs were washed in cold phosphate-buffered saline (PBS) and resuspended in complete RPMI. To obtain primary T cells, PBMCs were incubated overnight at 37 °C with 5% CO_2_. The following day, T cells in suspension were harvested and the adherent cells were discarded. Up to 70% of cells in the final culture were CD3^+^ T cells.

### Cells

Jurkat T cell line (clone E6.1) was obtained from ATCC and 293T cells from Cell Genesys (Foster City, CA). Jurkat cells and primary T cells were maintained in RPMI-1640 and 293T cells were maintained in Dulbecco’s modified Eagle’s medium. Media were supplemented with 10% heat-inactivated FCS, 2 mM Glutamax, 100 IU/ml penicillin, and 100 µg/ml streptomycin. Cells were grown at 37 °C in 5% CO_2_.

Cell viability was determined using a trypan blue exclusion method and counted using the Countess II FL automated cell counter (Invitrogen). 293F suspension cells, obtained from Thermo Fisher Scientific, were maintained in shaker flasks at 120 r.p.m., under 8% CO_2_ at 37 °C and propagated in their respective FreeStyle media (serum-free/defined).

#### LV expressing NS5A protein

HCV NS5A GT-1 sequence was obtained from pCMV NS5A TAG1 plasmid (Addgene, Plasmid #17646) and HCV NS5A GT-2 sequence was obtained from the J6/JFH infectious clone kindly provided by Dr. Charles Rice (Rockefeller University) [55]. FL HCV NS5A sequence for each GT was amplified and cloned into a modified pCIG3 vector (Addgene, Plasmid #78264) containing a C-terminal hemagglutinin (HA) tag. For expression of a short NS5A peptide, synthetic oligos with the restriction sites (*Bam*HI/*Eco*RI) were obtained from Integrated DNA Technologies (Coralville, IA) and directly cloned into pCIG3-HA vector. A point mutation in the NS5A protein was created with QuikChange Site-Directed Mutagenesis Kit (Stratagene, La Jolla, CA) following the manufacturer’s recommendation using primers containing the desired mutation. DNA sequences were confirmed by sequencing at the DNA core facility (CBER/FDA). Sequences of primers and oligos are provided in Supplemental Table 1.

#### LV production

LVs were manufactured in 293T cells using polyethyleneimine (PEI) transfection reagent (Polysciences). Briefly, 2 × 10^6^ cells were plated in 10 cm dishes and incubated overnight. The next day, the cells were washed with RPMI three times and replenished with 10 ml of fresh media. The transfection reaction mix was prepared with 36 µl of PEI, 6 µg of pCIG3-HA encoding HCV NS5A, 2 µg of vesicular stomatitis virus G envelope encoding plasmid, and 4 µg of the packaging plasmid pCD/NL-BH*DDD (a gift from Dr. Jakob Riser, FDA). Transfection complexes were gently mixed in 1 ml of OptiMEM (Thermo Fisher Scientific) and incubated for 15 min at room temperature. The mix was added dropwise to the cells and the dishes gently rocked. The next day, the media were changed, and supernatants were collected after 72 h. Supernatants were clarified (centrifugation at 1500 r.p.m. for 10 min) and concentrated using Amicon 100 kDa Centrifugal Filter Unit (Millipore). Final aliquots were then filter sterilized using a 0.45 µm filter. Concentrated vectors were stored at −80 °C. Vector transduction units were determined by transducing HEK293T cells and assessing GFP by flow cytometry.

#### HCV NS5A synthetic peptides

FITC-labelled synthetic peptides with an N-terminal HIV TAT protein transduction domain (TAT) alone (GGGGGRKKRRQRRR), or with the HCV NS5A (GT-1) a.a. 340–359 (GGGGGRKKRRQRRRHGCPLPPTKAPPIPPPRRKR), or the HCV NS5A (GT-2) (GGGGGRKKRRQRRRAGCALPPPKKAPTPPPRRRR) were purchased from GenScript Biotech. Peptides were dissolved in 100% dimethyl sulfoxide. Healthy donor T cells (1 × 10^6^ cells/ml) were incubated with 500 μg/ml peptide at 37 °C overnight, followed by washing twice with media before stimulation with 100 ng/ml of soluble anti-CD3. Cells were activated for 24 h in culture media containing 500 μg/ml of the peptide. Peptides were used at 500 μg/ml as preliminary experiments demonstrated that at this concentration synthetic NS5A peptides inhibited TCA without affecting cell viability. After 24 h, supernatants were collected and analyzed for IL-2 or IFN-γ.

#### Recombinant AAV vector production

AAV production was performed using Thermo Fisher 293F Freestyle suspension cell line and respective media under a modified triple transfection and double Iodixanol ultracentrifugation protocol. For transfection, cells were grown at 1 × 10^6^ cells/ml. A two plasmid AAV system utilizing pscAAV-GFP (CellBioLabs, #AAV-410) and pDGM6 (Addgene, #110660) was used in conjunction with PEI (molar ratio of 2 pDGMC:1 pscAAV-GFP for plasmids, along with a 2:1 PEI to DNA ratio). For expression of a short NS5A peptide, synthetic oligos with the restriction sites were obtained from Integrated DNA Technologies (Coralville, IA) and directly cloned into pscAAV-GFP vector. DNA sequences were confirmed by sequencing at the DNA core facility (CBER/FDA). Transfection complexes were carried out in OptiMEM and incubated for 15 min at room temperature and then applied to cells with fresh media. Following 72 h post transfection, cells were harvested, washed and resuspended in AAV cell lysis/stabilization buffer (0.001 P-F68, 200 mM NaCl, 200 mM MgCl_2_, 50 mM Tris-HCl pH 7.4 in dH_2_O), and then further lysed using a combination freeze/thaw (liquid nitrogen/37 °C water bath) and sonication. Sodium benzonase (final conc. of 50 U/ml), was added to the lysed cells for 1 h at 37 °C, which were then spun for 30 min at 10,000 r.p.m. to collect cellular debris. Clarified cell lysates were then loaded onto a discontinuous Iodixanol gradient (15, 25, 40, and 60% fractions prepared in PBS + MgCl_2_ and KCl). Samples were spun at 32,000 r.p.m. (SW32ti Beckman Coulter rotor) overnight. The viral fraction was then collected and diluted 1:1 in PBS for a second spin under identical conditions with the exception of a gradient change (30, 40, and 60% fractions). Following ultracentrifugation, the vector was passed through a 1-µm PES filter and then diluted in a Vector Wash Buffer (0.001 P-F68, 200 mM NaCl, 200 mM MgCl_2_, in PBS), for concentration and removal of Iodixanol using an Amicon 100 kDa PES cutoff column. The concentrated vector was then reformulated in AAV Resuspension Buffer (5% glycerol, 35 mM NaCl, 10 mM MgCl_2_ in PBS, filtered using 0.2 µM PES) and then aliquoted and stored at −80 °C. Vector titering was performed through a determination of transducing titers using serial dilutions in Jurkat cells for GFP expression and were expressed as TU/ml.

#### Transduction of cells

Jurkat cells were transduced with either control or HCV NS5A expressing LV at a multiplicity of infection (MOI) of 10. Following 24 h of transduction, cells were washed and GFP was analyzed at 72–96 h. GFP-expressing cells were bulk sorted using BD FACSAria (CBER Flow Cytometry Core) and expanded in culture for stimulation. Human T cells were transduced with an AAV vector at an MOI of 2.5. Following 48 h of transduction, cells were stimulated for 24 h before analysis.

#### Cell stimulation

Jurkat T cells (1 × 10^6^ cells/ml) were activated with plate-bound anti-CD3 (OKT3) and soluble anti-CD28 (BD) as described [27]. Isolated T cells were stimulated with soluble anti-CD3 (100 ng/ml). TCR-independent activation was measured following stimulation with PMA (50 ng/ml) and ionomycin (1 µg/ml). Following 24 h of stimulation, TCA was measured by assessing cytokines in the culture supernatant. To assess proximal TCR signaling, Jurkat cells were treated and stimulated with anti-CD3 (5 µg/ml) for 2 min. Activation of proximal TCR signaling was assessed by measuring phosphorylation of Lck, ZAP-70, and LAT by immunoblots.

#### ELISA

IL-2 or IFN-γ cytokines released in cell culture supernatant were quantified using human IL-2 or human IFN-γ ELISA Kits (BD Biosciences) according to the manufacturer’s instructions.

#### Immunoprecipitation

Cells were washed in PBS and lysed in lysis buffer (Pierce IP Lysis Buffer). Following cell lysis, lysates were clarified by centrifugation at 5000 r.p.m. for 5 min. Pellet was discarded, and the clarified lysate was mixed with respective antibodies and protein G magnetic beads (Dynabeads, Invitrogen) and incubated for 3 h at room temperature in a tube rotator. Beads were washed three times with PBS, and bound proteins were eluted by resuspending the beads and protein complex in Laemmli sample buffer and heated for 10 min at 95 °C. Beads were then separated using a magnet, and proteins in the sample buffer were subjected to immunoblot analysis. HCV GT-1 NS5A protein was immunoprecipitated using anti-NS5A (GT-1) antibodies (Abcam) and HCV GT-2 NS5A was immunoprecipitated using anti-HA antibodies that cross-reacted to the C-terminus HA-tag present in the NS5A (GT-2) protein. NS5A proteins were detected in immunoblots using two separate antibodies that were used in the immunoprecipitation.

#### Immunoblot analysis

Cellular lysates were mixed with Laemmli sample buffer, heated at 95 °C for 5 min and separated on NuPAGE Bis-Tris gels by electrophoresis and transferred to nitrocellulose membranes using the iBlot transfer system (Thermo Scientific). Membranes were incubated in previously heated 3% fat-free dry milk for 1 h at room temperature, followed by overnight incubation with primary antibodies. Proteins were detected with Super Signal West Dura (Thermo Scientific, #34075) using a Bio-Rad ChemiDoc MP imaging system. Immunoblots were quantified using ImageJ (NIH) by stripping and reprobing the same membrane for the indicated loading controls for normalization. Integrated Density (IntDen, the product of area and mean gray value) was calculated for each condition. In each experiment, the IntDen value was obtained for each condition and was normalized to the value obtained in the control stimulated LV condition using the following formula: (IntDen value/IntDen value at stimulated LV). Primary antibodies used were: pLck (Y394, R&D Systems), pZAP-70 (Y319), total Lck, total ZAP-70, total LAT and anti-HA from Cell Signaling, pLAT(Y226) from BioLegend, and β-actin and GAPDH from Sigma.

#### Statistics

One-way analysis of variance was used to compare results from multiple groups and a two-sided Student’s *t* test was used to compare results from two groups. *P* values <0.05 were considered statistically significant. GraphPad PRISM (GraphPad Software Inc.) was used for statistical analysis.

## Supplementary information

Supplemental Legends

Supplemental Figure 1

Supplemental Figure 2

Supplemental Table 1
