# Supplementary material for: A short hepatitis C virus NS5A peptide expression by AAV vector modulates human T cell activation and reduces vector immunogenicity
Source: Gene Ther. 2021 Nov 11;29(10-11):616–23. doi: 10.1038/s41434-021-00302-5 (PMC9091046; doi:10.1038/s41434-021-00302-5)
Supplement: Supplementary file 1 — Supplemental Legends [file 41434_2021_302_MOESM1_ESM.docx]

**Supplemental Figure 1:** GFP expression by Jurkat cells transduced with lentiviral vector expressing various HCV NS5A protein domains.

**Supplemental Figure 2:** Activation of proximal TCR signaling events was measured in Jurkat T cells transduced with a control LV or LV expressing the 20-mer NS5A (GT-1) peptide. Cell were either unstimulated (-) or anti-CD3 stimulated (2 minutes, +) and phosphorylation of Lck at tyrosine 394 (pY394), ZAP-70 at pY319 and LAT at pY226 was assessed. Total Lck, ZAP-70 and LAT were also measured and GAPDH was measured as a loading control. Experiment was repeated at least three times with similar results.

**Table 1:** Nucleotide sequence of primers and oligos used in the study.
