# Supplementary figures and images for: A short hepatitis C virus NS5A peptide expression by AAV vector modulates human T cell activation and reduces vector immunogenicity
Source: Gene Ther. 2021 Nov 11;29(10-11):616–23. doi: 10.1038/s41434-021-00302-5 (PMC9091046; doi:10.1038/s41434-021-00302-5)

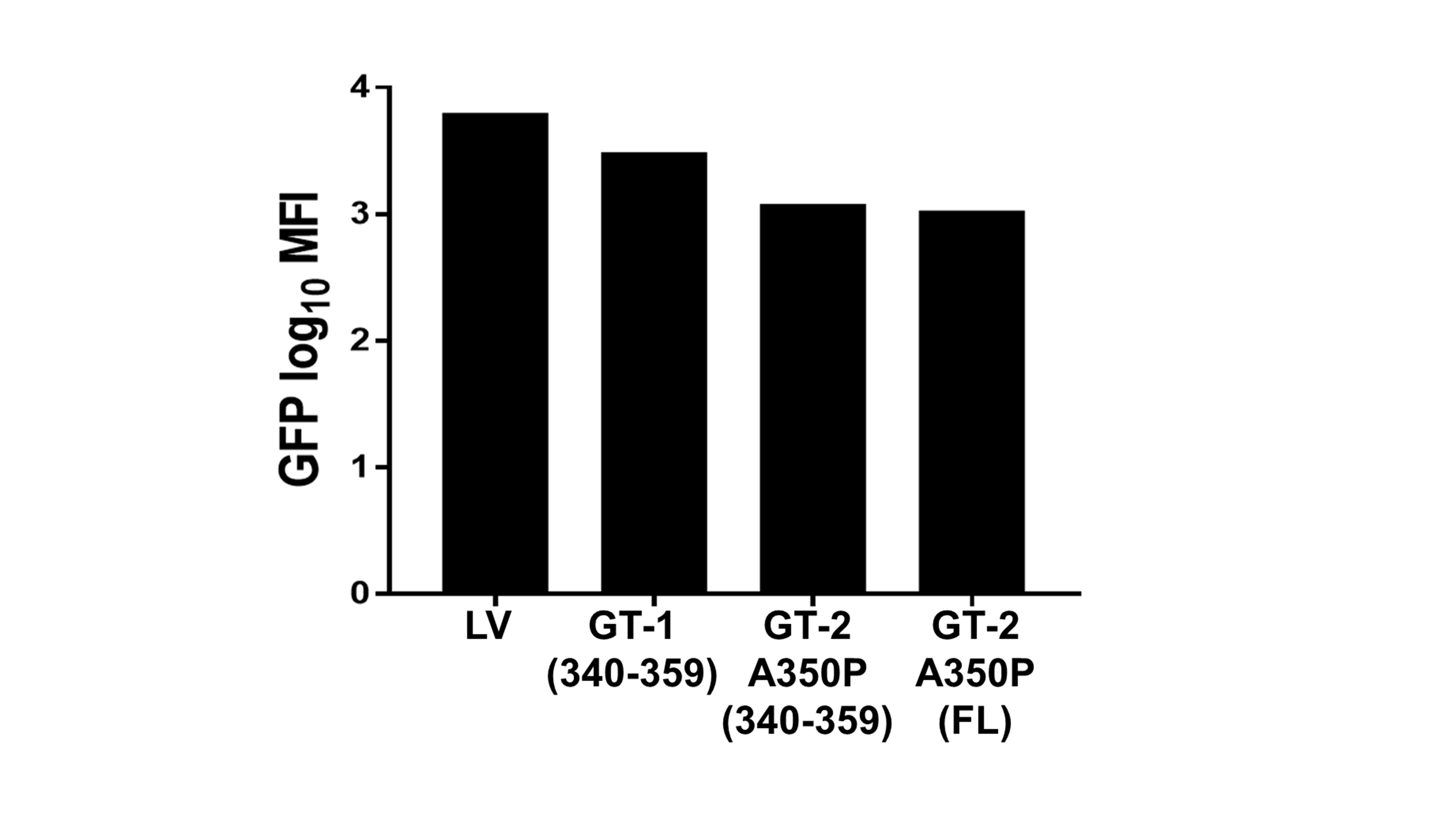

Supplement: Supplementary file 2 — Supplemental Figure 1 [file 41434_2021_302_MOESM2_ESM.tif]

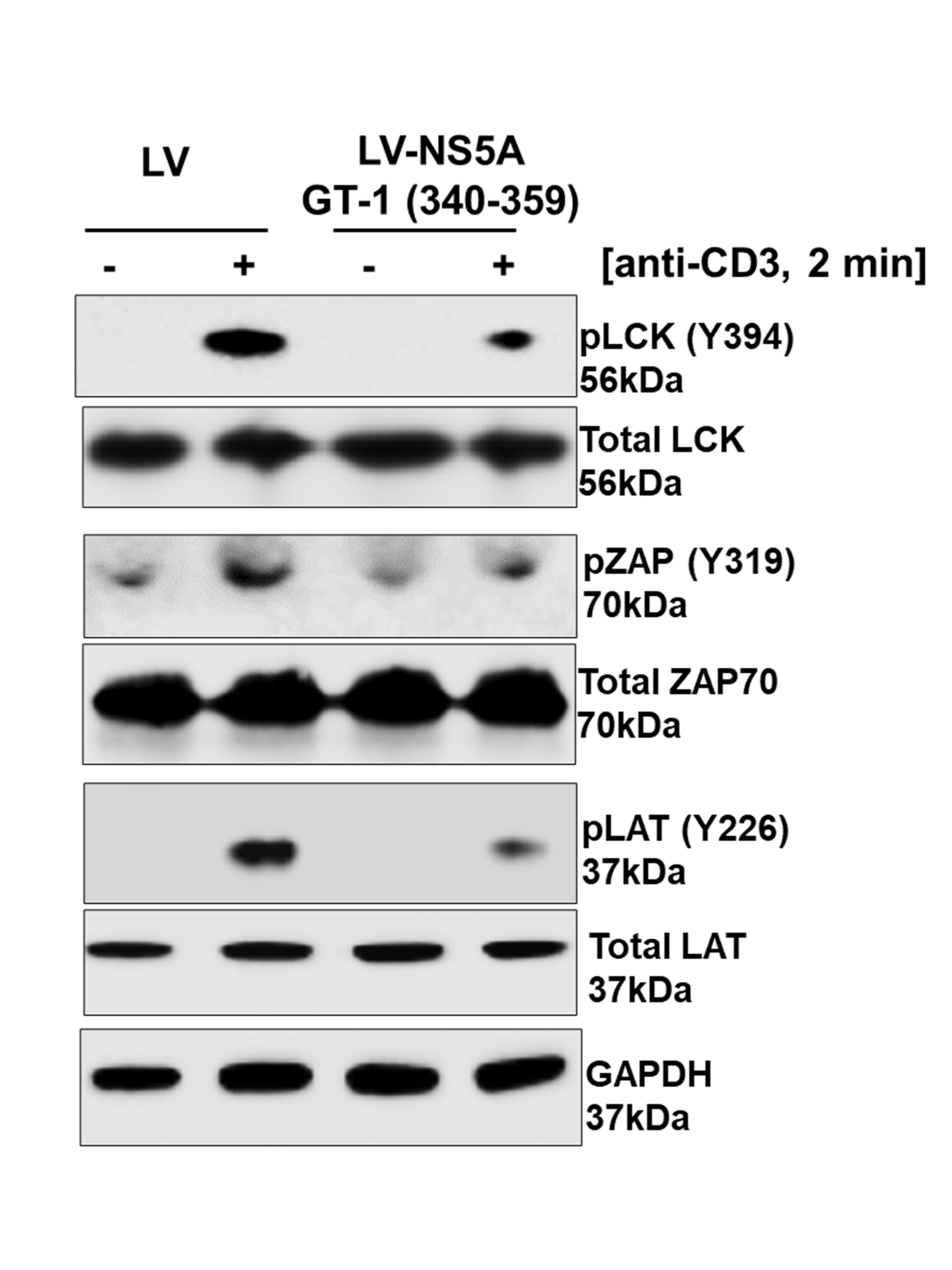

Supplement: Supplementary file 3 — Supplemental Figure 2 [file 41434_2021_302_MOESM3_ESM.tif]
