# Supplementary material for: A short hepatitis C virus NS5A peptide expression by AAV vector modulates human T cell activation and reduces vector immunogenicity
Source: Gene Ther. 2021 Nov 11;29(10-11):616–23. doi: 10.1038/s41434-021-00302-5 (PMC9091046; doi:10.1038/s41434-021-00302-5)
Supplement: Supplementary file 4 — Supplemental Table 1 [file 41434_2021_302_MOESM4_ESM.docx]

**Nucleotide sequence of primers and oligos:**

|  | **Sense (5’-3’)** | **Anti-Sense (5’-3’)** |
| --- | --- | --- |
| **HCV NS5A (GT-2)** | TAAGCAGCTAGCACCATGGCATCCGGATCCTGGCT | TGCTTAGAATTCGCAGCACACGGTGGT |
| **HCV NS5A 20mer (GT-1)** | GATCCGCCACCATGGCCCACGGATGCCCATTGCCACCTACCAAGGCTCCTCCAATACCACCTCCACGGAGAAAGAGGG | AATTCCCTCTTTCTCCGTGGAGGTGGTATTGGAGGAGCCTTGGTAGGTGGCAATGGGCATCCGTGGGCCATGGTGGCG |
| **HCV NS5A 20mer (GT-2) A346P** | GATCCGCCACCATGGCCGCTGGTTGTGCTCTCCCCCCCCCCAAGAAGCCCCCGACGCCTCCCCCAAGGAGACGCCGGG | AATTCCCGGCGTCTCCTTGGGGGAGGCGTCGGGGGCTTCTTGGGGGGGGGGAGAGCACAACCAGCGGCCATGGTGGCG |
| **A346P Mutagenesis Primer** | CAGCACCAGCGACAAGAAGAT | CCACGGGGATGGGAAAATACTT |
